# Vitamin D Supplementation at a Dose of 10 µg/Day in Institutionalized Children with Severe Motor and Intellectual Disabilities

**DOI:** 10.3390/nu16010122

**Published:** 2023-12-29

**Authors:** Yota Sato, Atsushi Kamei, Fumie Endo, Sakura Matsuyama, Hiroyuki Toda, Takeo Kasai

**Affiliations:** 1 Nutrition Support Team, Iwate Prefectural Rehabilitation and Nursery Center for Disabled Children, Iwate 028-3603, Japan; 2 Department of Pediatrics, Iwate Prefectural Rehabilitation and Nursery Center for Disabled Children, Iwate 028-3603, Japan; 3 Department of Developmental Disability Medicine, School of Medicine, Iwate Medical University, Morioka 028-3694, Japan

**Keywords:** nutritional support, severe motor and intellectual disabilities, ultraviolet exposure, vitamin D, vitamin D deficiency, vitamin D supplementation

## Abstract

Vitamin D (VD) deficiency can lead to health-related consequences. This study determined the effects of VD administration in VD-deficient children with severe motor and intellectual disabilities (SMID). Twenty-eight subjects were included. Among them, 25 subjects with parental consent for VD administration were given 10 µg/day (400 IU/day) of VD in April 2021. Serum 25-hydroxyvitamin D [25(OH)D] levels were measured at least 30 days after the start of VD administration. The total VD intake, serum 25(OH)D levels, and ultraviolet (UV) exposure before the blood tests were investigated. The results showed that the median serum 25(OH)D levels were 8.7 ng/mL (4.3–17.2) and 24.0 ng/mL (7.8–39 ng/mL) from March to May in 2020 and 2021, respectively. Among the 25 subjects, 22 with UV exposure had >20 ng/mL serum 25(OH)D level, and 2 without UV exposure had <20 ng/mL serum 25(OH)D level. Three subjects who did not receive VD supplementation had <20 ng/mL serum 25(OH)D level. Taken together, VD supplementation (10 µg/day) is effective in children with SMID in institutional care. Moreover, it may be sufficient for children with UV exposure, but not for those without.

## 1. Introduction

Vitamin D (VD) is obtained through dietary intake and cutaneous synthesis via ultraviolet (UV) exposure. Vitamin D deficiency (VDD) is a common metabolic/endocrine abnormality, and severe VDD causes hypocalcemia, rickets, and osteomalacia. Based on an expert consensus, VDD was defined as a serum 25-hydroxyvitamin D (25(OH) D) level of <20 ng/mL (50 nmol/L) and VD insufficiency as 20–30 ng/mL (50–75 nmol/L) [1]. Adequate sun exposure and dietary VD intake are important factors for preventing bone density loss and rickets. However, other factors such as extremely low antigravity exercise, use of antiepileptic drugs, and difficulty taking oral food from natural sources are also important in children with severe motor and intellectual disability (SMID) receiving institutional care. VD is an important factor for preventing osteoporosis and bone fractures in these children [2]. VDD is likely to occur in people living at high latitudes and indoors, those who excessively use sunscreen, those with pigmented skin, and those with insufficient ultraviolet (UV) B exposure [3]. Studies showed that VDD is common in the northern part of Japan because of limited daylight hours, especially during winter [4].

In our previous study, we reported that children with severe motor and intellectual disability (SMID) who had restricted UV exposure suffered from VDD despite meeting the Japanese recommended daily allowances (RDAs) of VD for each age and sex [5]. Children with SMID are at risk of bone mineral density loss due to lack of antigravity exercise, and measures should be taken to prevent bone mineral density loss. Although the notion that VD supplementation increases bone density has not been validated, maintaining an adequate VD blood level is important for bone health [6].

VD supplementation should be considered necessary for subjects with SMID, especially if they have reduced exposure to sunlight because of the full-time use of a ventilator. Kuwabara et al. reported a significant increase in the serum 25(OH)D levels of institutionalized elderly Japanese individuals treated with VD supplementation at a dose of 20 µg/day [7]. Previous studies in other countries have shown the benefits of VD supplementation with a dose of 20 µg/day in institutionalized elderly individuals [8,9]. The institutionalized elderly require 20 µg/day of VD supplementation [7,8,9]. However, to our knowledge, there are no studies of VD supplementation in institutionalized children with SMID. Therefore, the optimal VD dosage for children with long-term SMID has not been established [3]. The Japanese Dietary Reference Intakes for children under 15 years of age require 3–9.5 µg/day of VD [10], whereas the American Academy of Pediatrics recommends 10 µg/day from infancy to early childhood and beyond [11].

VDD is associated with asthma, diabetes, cancer, cardiovascular disease, and worsening musculoskeletal health in patients with cerebral palsy [12,13,14,15]. VD has been noted to have effects on the immune system, including autoimmune diseases and reactions to infections, including COVID-19 [16]. Therefore, the prevention of VDD is important for the health of children with SMID. VD intake through an adequate and balanced diet and supplementation should be recognized as one of the factors promoting the health of children with SMID. Moreover, achieving the appropriate blood levels of 25-hydroxyvitamin D [25(OH)D] should be recommended to help prevent health complications. However, there are no previous studies on VD supplementation in institutionalized children with SMID, and the appropriate dosage is unknown. In this study, the effects of 10 µg/day of VD supplementation in children with SMID admitted to a medical services facility were investigated, and serum 25(OH)D levels before and after supplementation were measured. Approximately half of the subjects were receiving VD supplementation that met the Japanese RDAs on dietary food and tube feeding intake before supplementation. About half of them were taking oral antiepileptic drugs to reduce VD levels.

## 2. Materials and Methods

### 2.1. Subjects (Table 1)

The current study included all children (*n* = 28) aged between 1 year and 3 months and 14 years and 10 months (median: 7 years and 8 months) who had been admitted at Iwate Prefectural Rehabilitation and Nursery Centre for Disabled Children from 1 April 2020 to 1 April 2021. Among them, 13 (46%) were boys and 15 were girls. In terms of feeding method, 14 were on complete tube feeding, 1 was on combined feeding, and 13 could intake orally. The underlying diseases included hypoxic–ischemic encephalopathy (*n* = 10), chromosomal abnormality (*n* = 3), multiple anomaly (*n* = 2), muscular dystrophy (*n* = 2), sequelae of encephalopathy (*n* = 2), schizencephaly (*n* = 1), hydrocephalus (*n* = 1), subdural hematoma (*n* = 1), congenital cytomegalovirus infection (*n* = 1), Angelman syndrome (*n* = 1), sequelae of bacterial meningitis (*n* = 1), sequelae of brain tumor (*n* = 1), spinal muscular atrophy (*n* = 1), and brain malformation (*n* = 1). As of 1 April 2020, the Gross Motor Function Classification System was III in 1 subject, IV in 6 subjects, and V in 21 subjects. Eight subjects used ventilators, and three required all-day ventilator therapy. Nine subjects were tracheostomized. Thirteen subjects took medication to reduce VD levels. Valproic acid was taken by nine subjects, and phenobarbital by six subjects (two in combination) [17]. One subject was taking a multivitamin (Panvitan^®^) (Teva Takeda Yakuhin Ltd., Nagoya, Japan) containing ergocalciferol (VD2).

None of the subjects were diagnosed with rickets, had repeated fractures, or had complained of bone pain. However, they could not have complained about pain because they were young and had SMID.

Among the 28 subjects, 25 (11 boys [44%]) with parental consent for VD supplementation were treated with 10 µg/day (400 IU/day) of BabyD200^®^ (Morishita Jintan Co., Osaka, Japan).

Iwate Prefectural Rehabilitation and Nursery Centre for Disabled Children is a residential facility that provides treatment and rehabilitation for children younger than 18 years with SMID. Children admitted to the center are unable to walk independently and require medical care and rehabilitation. The underlying medical conditions of the patients vary; moreover, many children require ventilators and tube feeding, as previously described. The center is located at 39.36° N, and subjects had no opportunity for UV exposure aside from going out or sunbathing in the courtyard because the windows of their rooms were not open.

**Table 1:** Subjects.

Group		Subject No.	Sex	Age (Years) †	Nutrition	Nutritional-Contents §	Diseases	Phase 2020			Phase 2021			UV before Phase 2020	UV before Phase 2021	VD Supplementation
Phase 2020	Phase 2021							Intake (μg/Day)	Ratio ‡	Serum 25(OH)D (ng/mL)	Intake (μg/Fay)	Ratio ‡	Serum 25(OH)D (ng/mL)			
α-2020	A-2021	1	F	3.25	Tube	Blended diet	HIE	4.5	1.13	4.3	15.1	3.78	23.3	◯	◯	◯
		2	F	3.4	Tube	Blended diet	Encephalopathy	3.2	0.8	4.3	14.6	3.65	21	◯	◯	◯
		3	M	4.1	Oral	Chopped soft diet	Schizencephaly	7.5	2.14	14.5	17.7	5.06	27.1	◯	◯	◯
		4	F	6.4	Tube	Blended diet, ENEVO^®^	HIE	8.0	1.6	17.2	18	3.6	29.3	◯	◯	◯
		5	F	8.5	Oral	Chopped soft diet	HIE	7.2	1.2	7.8	15.5	2.58	31.3	◯	◯	◯
		6	F	8.0	Oral	Regular diet	Encephalopathy	7.9	1.32	16.5	18.4	3.07	34.2	◯	◯	◯
		7	F	11.5	Oral	Regular diet	Muscular dystrophy	7.5	0.94	11.2	18	1.89	18	◯	◯	◯
		8	F	14.2	Oral	Chopped soft diet	Chromosomal abnormality	9.7	1.02	5.4	19.7	2.32	28.1	◯	◯	◯
		9	M	14.3	Oral	Regular diet	Muscular dystrophy	10.0	1.25	7	23.1	2.57	20	◯	◯	◯
		10	M	14.7	Oral	Regular diet	HIE	14.4	1.8	12.7	24.4	2.71	20.5	◯	◯	◯
	B-2021	11	M	11.4	Oral	Regular diet	Hydrocephalus	6.8	1.05	11.9	8.1	1.01	16.8	◯	◯	✕
β-2020	A-2021	12	M	1.25	Oral	Infant formula and baby food	Chromosomal abnormality	7.3	2.43	17	16.9	5.63	24.4	✕	◯	◯
		13	M	1.5	Tube	Blended diet	Subdural hematoma	2.7	0.9	4.8	13.1	4.37	31.8	✕	◯	◯
		14	F	4.5	Tube	Blended diet, RACOL-NF^®^	Multiple anomaly	4.1	1.03	6	15	3.75	37.7	✕	◯	◯
		15	F	6	Tube	Blended diet, ENEVO^®^	HIE	4.5	0.9	9.4	17.6	3.52	27.6	✕	◯	◯
		16	M	6.75	Oral	Blended diet	Multiple anomaly	6.6	1.47	7.3	16.7	3.71	38.4	✕	◯	◯
		17	F	7.1	Oral	Chopped soft diet	Congenital CMV infection	7.7	1.54	8.4	18.7	3.12	23.5	✕	◯	◯
		18	F	7.25	Tube	RACOL-NF^®^, Blended diet, V CRESC^®^	HIE	3.4	0.68	8.9	21.7	3.62	39	✕	◯	◯
		19	M	8.4	Oral	Chopped soft diet	Angelman syndrome	8.8	1.76	6	18.8	3.76	32.7	✕	◯	◯
		20	F	8.8	Tube	RACOL-NF^®^, V CRESC^®^	Chromosomal abnormality	4.4	0.73	13.2	21.9	3.65	30.3	✕	◯	◯
		21	F	11	Tube	ENEVO^®^, Blended diet	Bacterial meningitis	4.2	0.53	7.6	15.8	1.66	22.1	✕	◯	◯
		22	F	11.2	Tube	ENEVO^®^, RACOL-NF^®^	HIE	7.6	0.95	12.6	23.6	2.48	23	✕	◯	◯
		23	M	12	Combined	Blended diet, dysphagia food	Brain tumor	7.2	0.9	8.1	17.8	2.23	25.7	✕	◯	◯
		24	M	13.1	Oral	Regular diet	Spinal muscular atrophy	6.6	0.83	13.1	17.5	2.19	22.1	✕	◯	◯
	C-2021	25	M	2.75	Tube	Anti-regurgitation formula	HIE	2	0.67	10.9	12.5	3.57	16.8	✕	✕	◯
		26	M	4.2	Tube	Anti-regurgitation formula	HIE	7.9	2.26	15.2	19.8	5.66	19.1	✕	✕	◯
	D-2021	27	F	6.8	Tube	ENEVO^®^, Blended diet	HIE	2.7	0.54	6.7	9.1	1.82	9.1	✕	✕	✕
		28	M	14.8	Tube	Blended diet ENEVO^®^	Brain malformation	6.1	0.76	6.5	8.1	0.9	7.8	✕	✕	✕

HIE, hypoxic–ischemic encephalopathy; CMV, cytomegalovirus; 25(OH)D, 25-hydroxyvitamin D. † Age as of 1 April 2020. ‡ VD-intake-to-RDA ratio. § Nutritional content is listed in order of calories administered. In the table, ◯ indicates that the subject had the supplement or sun exposure opportunity, while ✕ indicates that there was not. ENEVO^®^ (Abbott Japan LLC., Tokyo, Japan), RACOL-NF^®^(Abbott Japan LLC., Tokyo, Japan), RACOL-NF^®^ (EN Otsuka Pharmaceutical Co., Ltd., Iwate, Japan), V CRESC^®^ (NUTRI Co., Ltd., Mie, Japan).

### 2.2. Examinations

This is a before and after study. Serum 25(OH)D was measured from May to June 2020 (Phase-2020) and from mid-April 2021. Among the 25 subjects, 10 µg/day of VD (BabyD200^®^) was administered. Serum 25(OH)D was measured at least 30 days after the start of treatment (May–June 2021 [Phase-2021]).

Blood was collected by venipuncture over 5 mL and serum was promptly obtained by centrifugation. Serum was kept under refrigeration at 5 °C or less until analysis. The measurements were performed by LSI Medience Corporation using the chemiluminescent enzyme immunoassay method. VD intake for 30 consecutive days was calculated before the blood test. In Phase 2021, VD intake was summed with the dose from supplements. For subjects on enteral nutrition, the daily VD intake was calculated from the daily enteral nutrition dosage per day over a 30-day period. For enteral nutrition, the calculation was based on the VD content of the package insert. For blended diet, the calculation was based on the VD content of the blended diet of our institution’s nutrition department. Similarly, oral intake was calculated from the VD content of the dietary prescriptions of the nutrition department. For subjects taking oral multivitamins, the intake from medication and dietary intake were combined. The RDAs by age and sex were based on the Dietary Reference Intakes for Japanese (2020) by the Ministry of Health, Labour and Welfare [10].

The actual intake/RDA ratio (intake/RDA ratio) was used as an indicator of intake per subject. The approximate intake per age and sex was based on age as of 1 April 2020 and 1 April 2021, respectively. Based on the guidelines for determining VD deficiency/insufficiency by the Japan Endocrine Society and Japanese Society for Bone Metabolism, VDD and VD insufficiency were defined as serum 25(OH)D level below 20 ng/mL and between 20 ng/mL and 30 ng/mL, respectively [18]. The half-life of 25(OH)D levels is approximately 15 days [19].

For this reason, we investigated whether the subjects attended school, went outdoors, and sunbathed within 15 days before Phase 2020 and Phase 2021 from their medical and leisure-time activity records.

### 2.3. Statistical Analyses

The serum 25(OH)D levels and intake/RDA ratios were analyzed using the Wilcoxon’s signed-rank sum test as a comparison of two groups with the non-parametric method, as it was a comparison of two samples from the same subject group. The comparison of the number of subjects with UV exposure opportunities before the blood test included nominal variables. Thus, the tχ^2^ test was used. The analysis of serum 25(OH)D levels and VD intake/RDA ratios with the non-parametric non-paired test was performed on group α-2020 and group A-2021. Therefore, the Mann–Whitney U test was used.

## 3. Results

### 3.1. Subjects and Grouping

**Figure 1:**
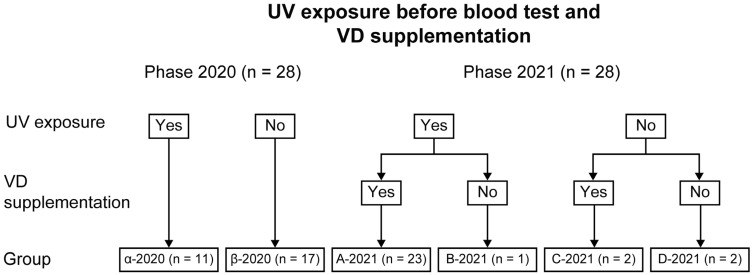
Grouping of subjects by UV exposure before Phase 2020 and Phase 2021 and VD supplementation before Phase 2021.

The subjects were allocated to one of two groups in Phase 2020 and four groups in Phase 2021 (Figure 1, Table 1).

In Phase 2020, the subjects were allocated to the following groups according to the opportunities for UV exposure before the blood test: Phase 2020 with (Group α-2020 [*n* = 11]) and without UV exposure (Group β-2020 [*n* = 17]). In Phase 2021, the subjects were allocated to the following groups: Group A-2021 with UV exposure before the blood test and VD supplementation (*n* = 23), Group B-2021 with UV exposure and no VD supplementation (*n* = 1), Group C-2021 with VD supplementation and no UV exposure (*n* = 2), and Group D-2021 without UV exposure and VD supplementation (*n* = 2). Based on the Phase 2020 blood test results, the number of subjects with UV exposure before the blood test increased from Phase 2020 to Phase 2021. This is because our institution has been working to increase UV exposure opportunities in our subjects whenever possible [5].

Two cases in Group B-2021 and Group D-2021 had no opportunities for UV exposure before Phase-2021 because of poor general condition or the need for full-time use of a ventilator.

### 3.2. VD Intake, UV Exposure, and Serum 25(OH)D per Phase

#### 3.2.1. Phase 2020

The serum 25(OH)D levels of subjects in Phase 2020 ranged from 4.3 ng/mL to 17.2 ng/mL, with a median value of 8.7 ng/mL. All 28 subjects had VDD (<20 ng/mL). Among them, 15 subjects had higher VD intake, whereas 13 subjects had lower VD intake than the RDA by age and sex within 30 consecutive days before Phase 2020. The intake/RDA ratio ranged from 0.53 to 2.43 (median: 1.03) (Table 1).

In Group α-2020 (*n* = 11) with UV exposure within 15 days before the blood test, their serum 25(OH)D levels and intake/RDA ratio ranged from 4.3 ng/mL to 17.2 ng/mL (median: 11.2 ng/mL) and 0.8–2.14 (median: 1.2), respectively.

In Group β-2020 (*n* = 17) without UV exposure within 15 days before the blood test, their serum 25(OH)D levels and intake/RDA ratio ranged from 4.8 ng/mL to 17.0 ng/mL (median: 8.4 ng/mL) and 0.53–2.43 (median: 0.9), respectively.

#### 3.2.2. Phase 2021

The serum 25(OH)D levels of subjects in Phase 2021 ranged from 7.8 ng/mL to 39.0 ng/mL with a median value of 24.0 ng/mL. Six subjects had VDD (<20 ng/mL), whereas fourteen subjects had VD insufficiency (20–30 ng/mL). VD intake within 30 consecutive days before Phase 2021 was higher than the RDA by age and sex in 27 subjects and lower in 1 subject. The intake/RDA ratio ranged from 0.9 to 5.66 (median: 3.32) (Table 1).

In Group A-2021 (*n* = 23) with UV exposure and supplementation within 15 days before the blood test, their serum 25(OH)D levels and intake/RDA ratio ranged from 18.0 ng/mL to 39.0 ng/mL (median: 27.1 ng/mL) and 1.66–5.63 (median: 3.52), respectively. One subject had VDD (<20 ng/mL), whereas 14 subjects had VD insufficiency (20–30 ng/mL).

In Group B-2021 (*n* = 1) with UV exposure within 15 days before the blood test and without VD supplementation, one subject had VDD with serum 25(OH)D level of 16.8 ng/mL and intake/RDA ratio of 1.01.

In Group C-2021 (*n* = 2) with VD supplementation and without UV exposure within 15 days before the blood test, both subjects had VDD with serum 25(OH)D levels of 16.8 and 19.1 ng/mL and intake/RDA ratios of 3.57 and 5.66.

In Group D-2021 (*n* = 2) without UV exposure and VD supplementation within 15 days before the blood test, both subjects had VDD with serum 25(OH)D levels of 9.1 and 7.8 ng/mL and intake/RDA ratios of 1.82 and 0.9.

### 3.3. Statistical Analysis

**Figure 2:**
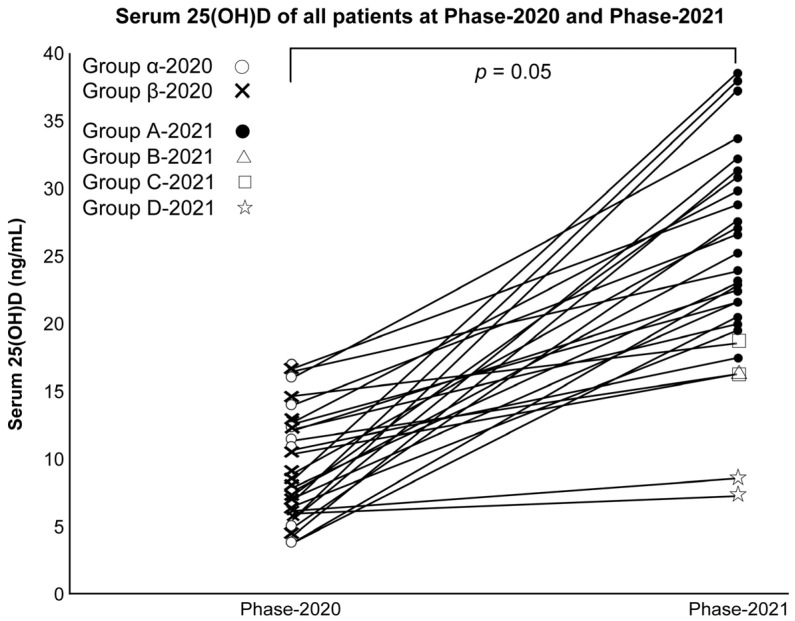
Serum 25(OH)D of all subjects in Phases 2020 and 2021.

Serum 25(OH)D and VD intake/RDA ratio increased significantly in all subjects in Phase 2020 and Phase 2021 (both *p* < 0.001; Wilcoxon’s signed rank sum test) (Figure 2).

Moreover, the number of subjects with UV exposure before the blood test increased significantly (*p* < 0.001; χ^2^ test).

**Figure 3:**
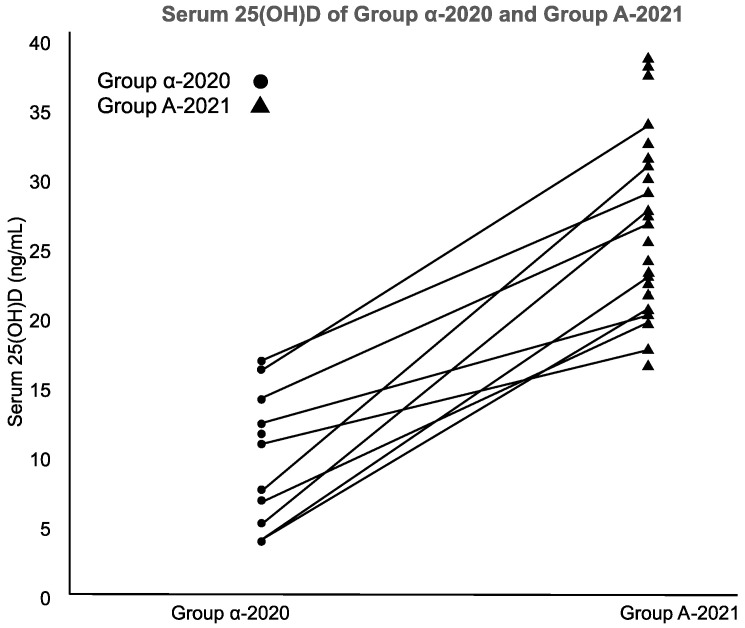
Serum 25(OH)D of Group α-2020 and Group A-2021.

Group α-2020 (*n* = 11) and Group A-2021 (*n* = 23) had opportunities for UV exposure but varying VD supplementation. Their serum 25(OH)D and VD intake/RDA ratio increased significantly (both *p* < 0.001; Mann–Whitney’s U-test) (Figure 3).

## 4. Discussion

This study presents a pioneering effort in addressing VDD among institutionalized children with SMID. Our findings showed that none of the 28 subjects in this study had serum 25(OH)D levels exceeding 20 ng/mL before VD supplementation, regardless of UV exposure. However, out of the twenty-five subjects who received VD supplementation, the serum 25(OH)D levels reached or exceeded ≥20 ng/mL in all subjects except in three of them and exceeded 30 ng/mL in eight subjects. Among the three subjects who did not achieve the target serum 25(OH)D levels of ≥20 ng/mL, two had no opportunity for UV exposure. In our previous study, institutionalized children with SMID were found to be severely deficient in VD levels because of limited UV exposure. This deficiency was observed even if subjects met the RDAs for age and sex as defined by Japanese standards [5]. Therefore, we recognized the need for VD supplementation and intervened. Within the framework of this study, we administered a daily supplement of 10 µg of VD to institutionalized children with SMID. In this study, the level of UV exposure was unlikely to be significant because of the spring season and limited amount of exposed skin. Even under these circumstances, the serum 25(OH)D levels were found to increase in UV-exposed subjects supplemented with 10 µg/day of VD, often exceeding 20 ng/mL. We solely assessed the presence or absence of daylight exposure and did not focus on factors such as frequency, duration, or extent of exposure. The results showed that 10 µg/day of VD was sufficient for children with potential UV exposure, but not for those with less UV exposure. Institutionalized children with SMID often have limited UV exposure because of the use of ventilators. The results of this study showed that 10 µg/day of VD supplementation was sufficient for most subjects to achieve adequate VD status even with limited UV exposure.

In Group C, two subjects without UV exposure had <20 ng/mL serum 25(OH)D levels even with 10 µg/day of VD supplementation. In institutionalized children with SMID, 10 µg/day would be inadequate if there is no opportunity for UV exposure. In Japanese institutionalized elderly with limited UV exposure, 5 µg/day of VD supplementation was reported to be ineffective, and only 41% of subjects exceeded 20 ng/mL serum 25(OH)D level even with 20 µg/day of VD supplementation [7,19]. The VD dose of 10 µg/day established in this study may not be sufficient for institutionalized SMID children with low sun exposure. Thus, setting an optimal dose that does not cause adverse effects is a subject for future research.

There is no consensus regarding the target serum 25(OH)D level. The Japanese Society for Bone and Mineral Research has considered 25(OH)D as an appropriate indicator of VD status and defined <20 and 20–30 ng/mL serum 25(OH)D level as deficient and insufficient, respectively, in terms of bone density and fracture risk [18]. This is the definition we adopted in this study. Frighi et al. have revealed that VDD is a cause of osteoporosis and fractures in people with intellectual disabilities and those who require treatment [20]. In their study, VD supplementation at a dose of 10 µg/day was not sufficient. Several patients who received supplementation at a dose of 20 µg/day achieved serum 25(OH)D levels of ≥20 ng/dL. Sakai et al. revealed that VDD and vitamin K deficiency are a cause of osteoporosis and fractures in institutionalized patients with SMID [21]. Children with cerebral palsy have a high incidence of VDD. Seth et al. reported that children with cerebral palsy, those with low UV exposure opportunities, and those using antiepileptic drugs have a high prevalence of VDD [22].

While there are various recommendations for the dosage of VD supplementation [23], the appropriate dosage for institutionalized children with SMID remains unclear [3]. In the Dietary Reference Intakes for Japanese, the RDAs for children in this study ranged from 3.0 µg/day to 9.5 µg/day [10]. This dose considers adequate cutaneous VD synthesis with UV exposure, which we previously reported as insufficient in institutionalized SMID children with limited opportunities for UV exposure [5]. The American Academy of Pediatrics recommends an intake of 10 µg/day from neonate to teenager [11]. This dose considers inadequate VD synthesis, such as darker skin pigmentation, and was adopted as the dose for children with SMID who may have limited UV exposure in the present study. The results of this study suggest that the resulting dose may be adequate when opportunities for UV exposure are available, but inadequate when they are not. Sakai et al. found that institutionalized patients with SMID had a significantly higher incidence of VDD than those receiving tube feeding [21]. Kuwabara et al. showed that insufficient vitamin doses or malabsorption in tube feeding could be a cause of vitamin deficiency [24]. Increasing VD doses may be considered in children with SMID who require tube feeding.

Although no subjects in this study experienced adverse events requiring discontinuation or dose reduction, with the highest 25(OH)D level at 39.0 ng/mL, 10 µg/day of VD supplementation was considered safe. However, the risk of overdosage should still be considered when administering VD supplements. Relatively high doses of VD supplements for infants (>40 µg/day for <1 year and >80 µg/day for 1–6 years) may cause hypercalcemia, hypercalciuria, clinical signs of vomiting, diarrhea, and irritability, but serious side effects are rare [25]. VD supplementation is considered as a potential source of new stones in subjects with idiopathic hypercalciuria and urinary tract stones. However, 10–20 µg/day of VD supplementation was reported to have no effect on serum calcium levels, urinary calcium excretion, or the formation of new urinary tract stones in children with idiopathic hypercalciuria [26]. In the present study, one subject had a history of urinary tract stones but did not develop new stones. According to the US Food and Nutrition Board, the adverse events of VD overdose include hypercalcemia, adverse renal and cardiovascular effects, increased incidence of cancer, and effects on all-cause mortality, and >50 ng/mL serum 25(OH)D level may be a risk factor for adverse events [27]. In the present study, dietary and supplemental VD intake did not exceed the age- and sex-specific tolerable upper limit (20–90 µg/day) [10], and no subjects had a 25(OH)D level >50 ng/mL. Assuming that the ideal 25(OH)D level is 30–50 ng/mL, the treatment of institutionalized children with SMID may need to be individualized, with 25(OH)D levels checked every few months and VD supplementation doses adjusted accordingly. In children with SMID, multiple causes are associated with reduced bone mineral density, including lack of antigravity exercise, VDD, and deficiencies in other nutrients. Hence, bone management is a challenging task [21]. This complexity also makes it difficult to measure the efficacy of interventions. Ideally, the effect should be a change in bone density or reduced fracture risk. However, this is challenging to assess as multiple causes are involved. In relation to this, we used serum 25(OH)D levels as an endpoint in this study.

### Limitation

Despite the findings, this study has some limitations. Serum 25(OH)D levels were measured using a chemical immunoassay. Mass spectrometry is the gold standard for measuring serum 25(OH)D levels. Using chemical immunoassays, the VD status of subjects might be underestimated [28].

The subjects were all Japanese, and the study was carried out in an institution for severely disabled children in the northern part of Japan. Therefore, the results may not be directly applicable to other ethnic groups or to other facilities located in districts with different latitudes. Nevertheless, the results provide important data on the importance and dosage of VD supplementation for children with SMID who have limited opportunities for UV exposure. It can provide a specific amount of evidence that may require further validation in multicenter studies, including those in other regions. Only the presence or absence of opportunities for UV exposure was assessed. The time and duration of UV exposure and the area of skin exposed were not considered. Ideally, the actual UV exposure should be measured using an ultraviolet dosimeter, but it is difficult to generalize [29]. To determine the risk of adverse events, the extent to which 25(OH)D increases during summer, when UV exposure is more intense, should be investigated. In addition, the sample size was a single-center study with a small size, especially for groups B, C, and D. VDD might have been an individual predisposition. Children with SMID who require all-day ventilator therapy are the most difficult to manage in terms of bone health, as VD synthesis in the skin is not expected and antigravity exercise is not possible. The strengths of this study include VD supplementation and the observed changes in serum 25(OH)D levels. Future studies should consider increasing the VD dose for children without UV exposure.

In this study, only the changes in serum 25(OH)D before and after vitamin D supplementation were assessed. We did not assess clinical changes such as changes in bone mineral density, fracture frequency or improvement in pain. Furthermore, the aim of improving VD status is to improve children’s current and future bone and general health. Thus, future studies should evaluate changes in bone mineral density and fracture in groups treated with VD supplements.

## 5. Conclusions

If institutionalized children with SMID are exposed to UV, VD supplementation at a dose of 10 µg/day is appropriate to prevent VDD; otherwise, the dosage is inadequate. In individuals without opportunities for UV exposure, increasing supplementation should be considered. Further, it is also important to take as many opportunities as possible for UV radiation exposure to prevent VDD.

## Data Availability

Data supporting the findings of this study are available upon request from the corresponding author. Data were not made publicly available due to privacy or ethical restrictions.
